# Structural Basis for D3/D4-Selective Antagonism of Piperazinylalkyl Pyrazole/Isoxazole Analogs

**DOI:** 10.3390/molecules30193917

**Published:** 2025-09-28

**Authors:** Kwang-Eun Choi, Seong Hun Jang, Woo-Kyu Park, Kyoung Tai No, Hun Yeong Koh, Ae Nim Pae, Nam-Chul Cho

**Affiliations:** 1 Drug Information Platform Center, Korea Research Institute of Chemical Technology, Daejeon 34114, Republic of Korea; 2 R&D Center, PharmCADD, Busan 331, Republic of Korea; 3 Division of Drug Discovery Research, Korea Research Institute of Chemical Technology, Daejeon 34114, Republic of Korea; 4 Department of Integrative Biotechnology, Yonsei University, Seoul 120749, Republic of Korea; 5 Department of Chemistry, Inha University, Nam-gu, Incheon 402751, Republic of Korea; 6 Center of Brain Disorders, Brain Science Institute, Korea Institute of Science & Technology, Seoul 02792, Republic of Korea

**Keywords:** D2-like dopamine receptors, subtype selectivity, 3D-QSAR, piperazinylalkyl pyrazole/isoxazole analogs

## Abstract

Dopamine D2-like receptors, including D2, D3, and D4, are members of the aminergic G protein-coupled receptor (GPCR) family and are targets for neurological disorders. The development of subtype selective ligands is important for enhanced therapeutics and reduced side effects; however, it is challenging to design and develop selective ligands owing to the high degree of sequence homology among D2-like subtypes. To gain insight into the structural basis of subtype selectivity of piperazinylalkyl pyrazole/isoxazole analogs for D2-like dopamine receptors, we carried out 3D quantitative structure–activity relationship (3D-QSAR) and molecular docking studies. The 3D-QSAR models for the D2, D3, and D4 subtypes showed robust correlation coefficients (r^2^) of 0.960, 0.912, and 0.946, as well as reliable predictive values (Q^2^) of 0.511, 0.808, and 0.560, respectively. Contour map analysis revealed key structural determinants for ligand activity, highlighting the distinct steric and electrostatic requirements for each subtype. These findings were further rationalized by molecular docking studies, which confirmed that interactions with non-conserved residues modulate binding affinity. Crucially, our analysis identified a critical structural basis for D4 subtype selectivity. This selectivity is attributed to a spatial constraint within the hydrophobic pocket formed by TMs 3, 5, and 6. This constraint restricts the orientation of bulky substituents on the 4-phenylpiperazine moiety. These findings provide actionable structural insights for the rational design of next-generation subtype-selective antagonists for D2-like dopamine receptors.

## 1. Introduction

Dopamine D2-like receptors belong to a subfamily of aminergic G-protein coupled receptors (GPCRs) and are subdivided into the D2, D3, and D4 subtypes. These receptors share high sequence identity within the transmembrane (TM) segments, with 72% for D2/D4, 73% for D3/D4, and 90% for D2/D3, respectively [1]. The biological functions of D2-like dopamine receptors, which couple with Gi/o proteins to inhibit adenylyl cyclase activity, have been associated with the development of neurological disorders such as schizophrenia, drug addiction, and Parkinson’s disease [2,3].

**Figure 1:**
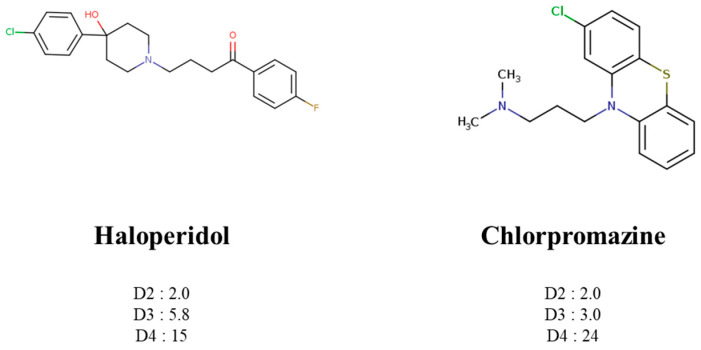
Representative antagonists of D2-like dopamine receptors. Activity values for D2, D3, and D4 receptors are expressed as K_i_ (nM) [34].

To treat schizophrenia via the dopamine receptors [4], the development of subtype-selective ligands is required to improve therapeutic efficacy and reduce side effects. Generally, antipsychotics such as haloperidol and chlorpromazine have been used to inhibit the activation of the dopamine D2 receptor for schizophrenia [5,6,7,8], but they also cause serious side-effects, such as extrapyramidal syndrome, tardive dyskinesia, and neuroleptic malignant syndrome (Figure 1) [9]. Clozapine, which has a high affinity for the D4 subtype, has been shown to be an effective therapeutic that reduces schizophrenic symptoms with minimal extrapyramidal syndrome [10,11]. This discovery has led to the development and design of subtype-selective drugs targeting dopamine D2-like receptors [12,13]. Although there is controversy regarding the primary subtype of dopamine receptors involved in the development of schizophrenia [14], subtype-selective ligands have been developed and have shown promise for the treatment of schizophrenia and drug addiction (e.g., FAUC213 [15] for D4 subtype, SB-277011A [16] R-22 [17] and NGB 2904 [18] for D3 subtype). However, it remains difficult to design and develop subtype-selective ligands because of the high degree of sequence homology of dopamine D2-like receptors [1,19].

In 2010, the crystal structure of the human D3 dopamine receptor was reported [20], which facilitated the elucidation of structure–activity relationships (SARs) in dopamine receptor subtypes. Subsequently, many researchers have reported the subtype selectivity determinants for D2-like dopamine receptors. Among diverse chemical moieties, the 4-phenylpiperazine series is well characterized and serves as a privileged scaffold for targeting aminergic GPCRs [21,22,23]. To enhance the affinity and selectivity of the D3 subtype over the D2 subtype, the 4-phenylpiperazine is connected to an aryl amide group via a flexible butyl linker (reviewed in ref. [24]), and the chemical diversity of the aryl amide group plays a pivotal role in the pharmacological specificity of the D3 receptor over the D2 receptor [16]. Based on the structure of dopamine receptors, the secondary binding pocket composed of transmembrane (TM) 1, 2 and 7 serves as the primary recognition site of the butyl amide group, where structural changes may occur at inter-helix contacts between TM2/TM7 and TM1 bundles, particularly involving the non-conserved residues of TM1 of the dopamine D3 receptor, with the spatial position of Tyr36^1.39^ of the D3 subtype [20,25]. Indeed, chimeric studies have revealed that diverse extracellular loop (ECL) 2 and 1 have contributed to ligand binding and subtype selectivity on the D3 versus D2 subtypes [26,27].

In mutagenesis studies, key residues were clearly identified for enhancing the selectivity of the D4 subtype because of the heterogeneous sequence of amino acids for the D4 subtype compared to the D2/D3 subtypes. Most notably, five residues (Leu2.60, Phe2.61, Ser2.64, Leu3.28, and Met3.29) may commonly explain the ligand binding specificity on the D4 subtype [28,29,30]. Additionally, several researchers [28,29] reported that elongation of the aryl chain of 4-phenylpiperazines changes the binding affinity of the ligand from the D4 to the D2/D3 subtype. In other words, 4-phenylpiperazines with short aryl linkers are preferable for D4 subtype affinity over D2/D3 subtypes, but the reason for this dependence of ligand binding affinity on linker length for the D4 subtype remains unclear.

Recently, numerous computational and experimental studies on GPCRs have been conducted [31,32], including investigations of the DR1 receptor [33], as well as the D3 [34] and D2 receptors [35]. However, these efforts have primarily relied on docking-based approaches, with limited integration of QSAR methodologies. Notably, no comprehensive studies combining docking and QSAR analyses have been reported for the selective D4 receptor.

Over the past decade, we have developed and synthesized a piperazinylalkyl pyrazole/isoxazole library targeting dopamine D3 and D4 receptors to aid in the discovery of antipsychotic drugs [36]. In this study, we focused on the structural basis of piperazinylalkyl pyrazole/isoxazole analogs for the subtype selectivity of D2-like dopamine receptors using computational methods.

## 2. Results and Discussion

### 2.1. 3D-QSAR of Piperazinylalkyl Pyrazole/Isoxazole Analogs for the Activity of D2/D3/D4 Subtypes

**Figure 2:**
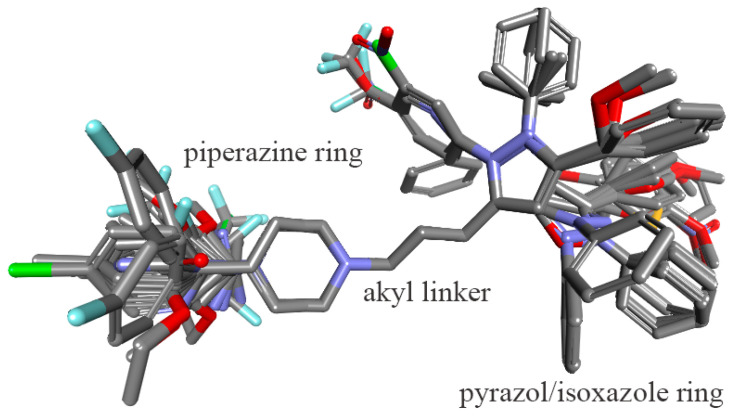
Molecular alignment of 141 piperazinylalkyl pyrazole/isoxazole analogs based on maximum common substructure.

To ensure reliable model construction, all compounds were first converted to 3D structures and energy-minimized at physiological pH levels. The protonation states were verified at pH 7.4 (Supplementary Figure S1). Molecular alignment was performed using the maximum common substructure, N,N-dimethylpropan-1-amine, as a reference framework (Figure 2). This alignment strategy allowed for consistent overlay of the pyrazole/isoxazole and 4-phenylpiperazine moieties, thereby minimizing conformational bias across the training and test sets and ensuring that steric and electrostatic field descriptors were meaningfully comparable.

**Figure 3:**
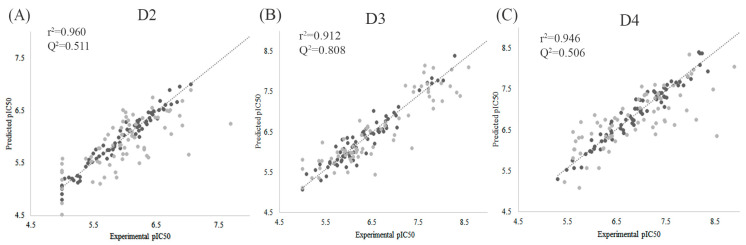
Correlation plots between the experimental value and predicted value of 3D-QSAR models for the intrinsic activity of D2 (**A**), D3 (**B**), and D4 (**C**) subtypes. ●: Training set; ●: Test set.

Using this aligned dataset, we developed 3D-QSAR models to investigate the structural determinants of subtype activity and selectivity for dopamine D2-like receptors. A total of 141 piperazinylalkyl pyrazole/isoxazole analogs were divided into a training set (n = 71) and a test set (n = 70) (Supplementary Table S1). To ensure representativeness, we first examined the chemical space distribution at the 2D level within the training and test sets, confirming that both sets were well balanced (Supplementary Figure S2). The partial least squares (PLS) regression analysis yielded statistically reliable models with non-cross-validated correlation coefficients (r^2^) of 0.960, 0.912, and 0.946 for the D2, D3, and D4 subtypes, respectively (Table 1 and Figure 3; see details in Supplementary Table S2). Predictive performance was further supported by Q^2^ values of 0.511, 0.808, and 0.560, demonstrating acceptable external predictivity, particularly for the D3 subtype. Although the D2 model exhibited a modest q^2^ value (0.476), this can be attributed to the relatively narrow activity range of compounds for this receptor subtype. Selected compounds with structures and pIC_50_ values are listed in Table 2.

### 2.2. Graphical Interpretation of Contour Maps of 3D-QSAR Models

**Figure 4:**
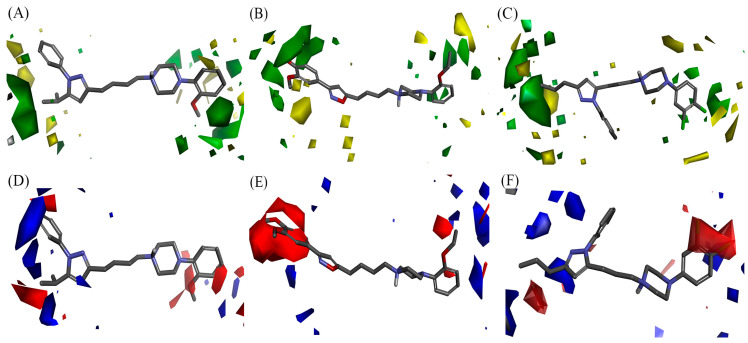
Contours of 3D-QSAR models with highly potent compounds for D2, D3, and D4 subtypes. The steric (**A**–**C**) and electrostatic contour (**D**–**F**) maps are shown together with highly potent compounds **32** (**A**,**D**), **130** (**B**,**E**), and **9** (**C**,**F**) for the D2, D3, and D4 subtypes, respectively. The green contour indicates regions where sterically bulky groups increase inhibitory activity, while the yellow contours indicate regions where sterically bulky groups decrease inhibitory activity. The blue contour indicates regions where electropositive charged groups increase inhibitory activity, while the red contour represents where electronegative charged groups improve inhibitory activity.

Contour map analysis provided clear insights into steric and electrostatic contributions (Figure 4)**.** For D2, bulky substituents at the 5-position of the pyrazole and ortho-/meta-positions of the 4-phenylpiperazine ring enhanced activity, with electronegative substituents at these sites further supporting potency. For D3, steric and electronegative substituents at the 3,4-positions of the phenylisoxazole ring and electropositive substituents at the ortho-position of the 4-phenylpiperazine ring were favored, while a neighboring yellow contour indicated steric restrictions in the receptor pocket. Supplementary Figure S3 further highlights that the linker length modulates substituent orientation: pyrazolyl-butyl derivatives (e.g., compound **88** with a 5-methylmethoxy substituent) occupy the green steric region more efficiently and thus display higher potency compared with their propyl analogs (e.g., compound **78**). For D4, bulky substituents at the 5-position of the pyrazole and negatively charged substituents at the ortho-/para-positions of the 4-phenylpiperazine were strongly favorable, consistent with the high activity of halogenated analogs (e.g., compounds **9**, **21**, **22**, and **46**). Supplementary Figure S4 additionally reveals scattered blue and green contours around the N1- and 5-positions of the pyrazole, indicating that dual substitution with electropositive bulky groups in these regions can synergistically enhance D4 activity. Moreover, a large red contour surrounding the para-position of the 4-phenylpiperazine ring emphasizes the advantage of halogen substituents, such as dichloro groups in compound **9**, which significantly improve binding affinity compared with unsubstituted analogs.

While these models effectively explain the intrinsic activity for each subtype, understanding the structural basis for selectivity requires a more direct comparison. Therefore, to elucidate the specific molecular features driving D4 preference over other subtypes, we generated additional 3D-QSAR models for the D4/D2 and D4/D3 ratios (Supplementary Results; Supplementary Table S3). These models exhibited strong statistical performance (r^2^ = 0.872 and 0.952; q^2^ = 0.478 and 0.752 for D4/D2 and D4/D3, respectively), with predicted selectivity values aligning with experimental observations (Supplementary Figure S5). Contour analyses (Supplementary Figure S6) revealed that the 4-phenylpiperazine moiety is the principal determinant of D4 selectivity. Bulky, negatively charged groups at the meta-/para-positions enhanced D4 preference, although only within a limited steric window; excessively bulky substituents (e.g., CF_3_, diphenylmethyl) reduced D4 activity (compounds **25**, **49**, **50**, **93**, and **103**). Selectivity also depended on pyrazole substitution: bulky groups at the N1-position favored D4/D2 selectivity, whereas substituents at the 5-position favored D4/D3 selectivity, which is in agreement with the superior selectivity of N1-phenyl-substituted pyrazoles (compounds **29**–**38**) compared with N2-substituted analogs (compounds **16**–**28**).

In summary, by integrating intrinsic activity and selectivity models, the 3D-QSAR analysis establishes that D4 subtype preference is dictated by a balance between steric effects at the pyrazole moiety and electrostatic optimization of the 4-phenylpiperazine substituents. Together with the molecular alignment strategy, these findings provide a coherent structural rationale for subtype selectivity and offer practical guidelines for the rational design of D4-selective antagonists.

### 2.3. Structural Requirement for Ligand Binding on Dopamine D2/D3/D4 Receptors

**Figure 5:**
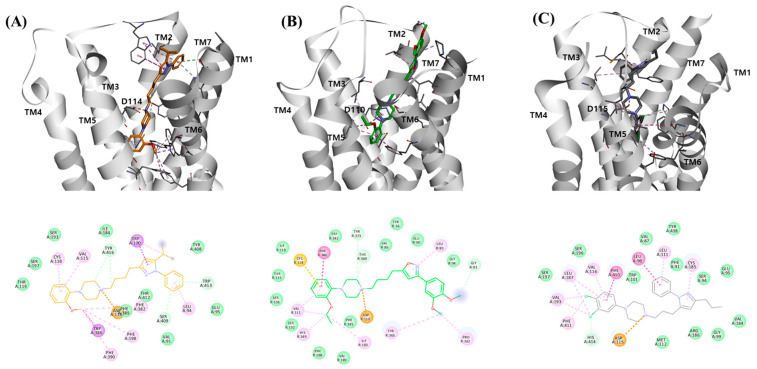
Docking poses of highly potent compounds on dopamine D2, D3, and D4 receptors. Binding modes (up) with 2D diagrams (down) on compound **32** for D2 ((**A**), brown), **130** for D3 ((**B**), green), and **9** for D4 subtypes ((**C**), grey) are represented. Hydrogen bonds, hydrophobic interactions, and charge interactions are shown with green, pink, and orange dotted lines, respectively. The interaction residues are drawn with a white line.

To further validate the structural insights derived from the 3D-QSAR analysis, we performed molecular docking studies using the crystal structures of the D2 (PDB ID: 6CM4), D3 (PDB ID: 8IRT), and D4 (PDB ID: 5WIV) receptors (Figure 5). Docking was carried out with representative high-affinity ligands for each subtype, and the resulting binding modes were compared with the steric and electrostatic features predicted by the 3D-QSAR models. The binding mode of compound **32** showed that a hydrophobic interaction between the pyrazole ring and Trp100^EL1^ of the D2 subtype encouraged a similar hydrophobic interaction between the phenyl and *i*-propyl substituents of the pyrazole ring with hydrophobic residues in the secondary binding pocket involving Trp100 ^EL1^, Trp413^7.37^, Leu94^2.64^, and Glu95^2.65^. This ligand pose was consistent with the steric and electrostatic contours of the 3D-QSAR model for D2 subtype, which displayed blue contours at the N1-position of the pyrazole moiety and both green and red contours at the 5-position of the pyrazol ring. In the hydrophobic pocket comprising TM3/TM5 and TM6 in the D2 subtype, the *t*-amine of the piperazine ring formed a charge interaction with Asp114^3.32^ residue, and the phenyl ring deeply occupied a pocket surrounding Val115^3.33^, Ser193^5.42^, Ser197^5.46,^ and Phe389^6.51^, with the position of the *o*-methoxy substituent extending into the extracellular region.

In the D3 subtype, the binding mode of compound **130** showed stabilization of the isoxazole ring via interactions with Leu89^2.64^, orienting the 3,4-dimethoxy substituents of the phenylisoxazole toward the solvent-exposed pocket formed by TM1, TM2, and TM7. This placement corresponds well with the large red and green contours identified in the QSAR model (Figure 4B and Supplementary Figure S3), which predicted that bulky electronegative substituents at the 3,4-positions enhance affinity. Additionally, the o-ethoxy substituent of the 4-phenylpiperazine moiety engaged hydrophobic residues Ser192^5.42^, Ser196^5.46^, Phe345^6.51,^ and His349^6.55^, consistent with the steric tolerance indicated by the yellow contour in the D3 model.

For the D4 subtype, the docking of compound **9** revealed that the 5-n-propyl substituent of the pyrazole moiety fit deeply within the hydrophobic pocket formed by TM3, TM5, and TM6, which is in agreement with the favorable green steric contour predicted in the 3D-QSAR model (Figure 4C and Supplementary Figure S4). The *m*, *p*-dichloro substituents of the 4-phenylpiperazine further contributed to hydrophobic interactions with Val116^3.33^, Ser197^5.43^, Ser200^5.46^, Phe410^6.51^, and His414^6.55^, while maintaining the essential salt bridge with Asp115^3.32^. These features directly reflect the large red electrostatic contours at the ortho- and para-positions of the 4-phenylpiperazine identified in the QSAR analysis, which emphasized the importance of electronegative substituents for D4 activity.

Taken together, the docking studies corroborate the 3D-QSAR predictions by demonstrating that the steric and electrostatic requirements identified in contour maps are realized in specific ligand–receptor interactions. The complementary agreement between the two approaches strengthens the conclusion that D4 selectivity is governed by steric accommodation within the hydrophobic pocket, as well as the electrostatic optimization of 4-phenylpiperazine substituents.

## 3. Materials and Methods

### 3.1. Dataset

All compounds and their biological activity data (IC_50_ values) against the D2, D3, and D4 receptors were taken from our previously published studies [37,38,39,40]. A total of 141 compounds were evaluated in experimental tests for their inhibitory activity (IC_50_ values) against D2-like dopamine receptors. The two-dimensional structure of the compounds is listed in Supplementary Table S1. To generate 3D-QSAR models, the dataset was divided into a training set (*n* = 71) and a test set (*n* = 70) based on ten molecular properties, ensuring a balanced representation of each receptor subtype in the test set and enabling a more rigorous evaluation of predictive performance: ALogP, Molecular_Weight, Num_Atoms, Num_Rings Num_H_Donors, Num_H_Acceptors, Num_RotatableBonds, Num_AromaticRings, Num_Fragments, and Molecular_PolarSurfaceArea. Given that these receptor subtypes exhibit structural differences that can influence ligand-binding characteristics, we considered it essential to employ a relatively large test set to validate the predictive capacity of our models across all three receptors. In addition, principal component analysis (PCA) was performed using Extended-Connectivity Fingerprints (ECFP6) and Functional-Class Fingerprints (FCFP6), which were generated using RDKit 2025.03.6.

### 3.2. Molecular Alignment

All compounds were first sketched as 2D structures and then converted into 3D structures. The compounds were protonated at pH 7.4, with the protonation state of piperazine verified [41] and QSAR analyses performed in the protonated state [42], followed by energy minimization using the CHARMM force field [43] in DiscoveryStudio 3.1 (Accelrys Inc., San Diego, CA, USA). The best alignment of compounds was obtained via an overlap of the maximum common structure (N, N-dimethylprorpan-1-amine) using Pipeline Pilot, version 8.1 (Accelrys Inc., San Diego, CA, USA).

### 3.3. Building 3D-QSAR Models

The 3D-QSAR models were built using the Create 3D-QSAR Model module in DiscoveryStudio. The pIC_50_ (−logIC_50_) values were used as the dependent variables in the 3D-QSAR calculations. The partial charges of all atoms were assigned with a CFF force field [44]. The steric and electronic fields were computed on a grid with a spacing of 1.5 Å, using a +1.0 point charge as the electrostatic potential probe, a carbon atom with a 1.73 Å radius as the van der Waals potential probe, and the distance-dependent dielectric constant to simulate solvation effects. The partial least squares (PLS) regression method [45] was used to construct a linear correlation between the 3D field (independent variable) and the biological activity values (dependent variable). All 3D-QSAR models in this study were evaluated via a five-fold cross-validation of the internal training set, which yielded the optimal number of components and the lowest standard error of prediction and cross-validation coefficients (q^2^). Then, the optimal number of components was employed to build 3D-QSAR models, using non-cross-validation to obtain the conventional correlation coefficient r^2^ and standard deviation. The predictive performance of the models was evaluated using the prediction of the biological activity of 70 compounds as an external test set (Q^2^).

### 3.4. Molecular Docking

All ligands were prepared using LigPrep ver. 2.5 in Maestro (Schrödinger, LLC, New York, NY, USA, 2012). This module conducts energy minimization, protonation at pH 7.4, and the generation of stereoisomers. Molecular docking was performed using the docking program GOLD Suite ver. 5.1 with the GOLD fitness function (CCDC, Cambridge, UK). Ligands and proteins were set to “flexible” and “rigid”, respectively. A distance constraint was applied between the tertiary amine of the piperazine ring and the carboxylate oxygen of the conserved Asp in TM3 (D114^3^·^32^, D110^3^·^32^, D115^3^·^32^ for D2, D3, and D4, respectively). The docking pose of the compound was selected according to its high GOLD scoring function.

## 4. Conclusions

In this study, we systematically investigated the structural determinants of subtype activity and selectivity of piperazinylalkyl pyrazole/isoxazole analogs at dopamine D2-like receptors using a combination of 3D-QSAR modeling and molecular docking. The 3D-QSAR models for D2, D3, and D4 subtypes demonstrated robust statistical quality and predictive performance, enabling reliable interpretation of steric and electrostatic contour maps. These models revealed that activity enhancement depends on distinct substituent requirements across the subtypes: bulky and electronegative substituents at the 5-position of the pyrazole and at ortho-/para-positions of the 4-phenylpiperazine for D4, steric and electronegative groups at the 3,4-positions of the phenylisoxazole for D3, and combined steric/electrostatic features in both the pyrazole and 4-phenylpiperazine moieties for D2.

Selectivity models further clarify that D4 preference arises from sterically constrained yet electrostatically favorable substitutions. Specifically, the N1-position of the pyrazole is critical for D4/D2 selectivity, whereas the 5-position governs D4/D3 selectivity, which is in agreement with experimental SAR trends. Molecular docking corroborates these findings by showing that the predicted steric and electrostatic hotspots are realized in concrete ligand–receptor interactions within hydrophobic and electrostatic pockets, particularly involving Asp^3.32^ and non-conserved residues in TMs 2–3 and 5–6.

In conclusion, our integrated computational analysis establishes that D4 subtype selectivity is governed by a synergy between steric accommodation at the pyrazole moiety and electrostatic optimization of the 4-phenylpiperazine substituent. These interactions occur within a structurally constrained binding pocket, providing a clear molecular basis for selectivity. Importantly, this study represents the first structural investigation of our synthesized piperazinylalkyl pyrazole/isoxazole analogs, as well as the first comprehensive analysis highlighting subtype-selective determinants for the dopamine D4 receptor [36,37,38,39,40]. These results not only offer a coherent structural rationale for the observed structure–activity relationship (SAR) trends but also provide practical and actionable design principles for the development of novel, highly selective D4 receptor antagonists.

## Figures and Tables

**Table 1:** Summarized statistics of 3D-QSAR models for the activity of D2, D3, and D4 subtypes.

PLS Statistics	D2	D3	D4
r^2^	0.960	0.912	0.946
r^2^ RMS residual error	0.107	0.2056	0.1682
q^2^	0.476	0.677	0.618
q^2^ RMS residual error	0.394	0.397	0.449
Number of components	6	3	4
Q^2^	0.511	0.808	0.560
Q^2^ RMS error	0.423	0.426	0.550
Mean absolute error	0.327	0.322	0.414

r^2^ is the non-cross-validated regression coefficient; q^2^ is the five-fold cross-validated regression correlation coefficient; Q^2^ is the cross-validated correlation coefficient of the test set; RMS is the root mean square.

**Table 2:** Structure of selected compounds with pIC_50_ values for D2/D3 and D4 subtypes.

Num.	Structure	D2	D3	D4	Num.	Structure	D2	D3	D4
**7**	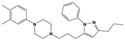	6.33	5.28	8.23	**81**	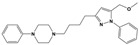	5.11	6.34	6.29
**8**	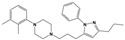	7.06	6.16	8.46	**84**	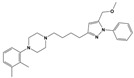	6.08	7.07	6.39
**9**	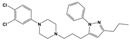	6.88	5.69	8.89	**86**	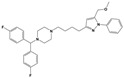	5.66	6.75	6.04
**18**	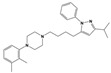	6.45	6.70	7.12	**88**	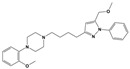	5.94	7.08	6.28
**19**	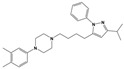	5.99	5.97	7.53	**96**	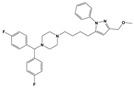	6.04	7.04	6.17
**21**	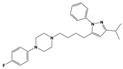	5.72	5.74	7.52	**103**	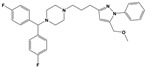	5.48	6.10	5.30
**22**	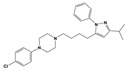	5.55	6.05	7.61	**109**	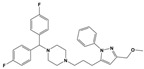	5.60	7.37	5.44
**29**	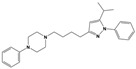	6.00	6.52	6.63	**112**	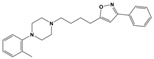	5.60	7.92	7.02
**32**	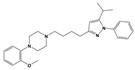	7.69	6.99	7.26	**121**	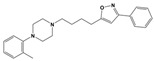	5.19	6.53	6.17
**39**	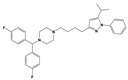	6.09	6.82	5.99	**124**	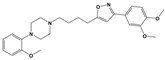	6.38	8.28	6.59
**46**	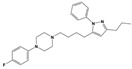	5.55	5.83	8.01	**130**	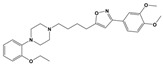	6.92	8.59	7.55
**51**	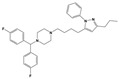	6.20	5.85	7.40	**137**	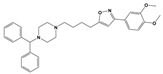	5.58	7.70	5.61
**55**	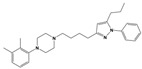	6.22	7.02	7.31	**140**	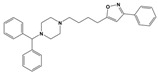	6.3	7.8	5.6
**78**	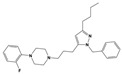	6.06	5.00	6.94	**141**	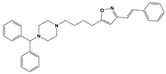	6.31	7.40	6.22

## Data Availability

All data generated or analyzed during this study are included in this published article and its supplementary information files.

## Supplementary Materials

The following supporting information can be downloaded at https://www.mdpi.com/article/10.3390/molecules30193917/s1: Supplementary Table S1: The structure of compounds for training and test set; Supplementary Table S2: The experimental values and predicted values of 3D QSAR models.; Supplementary Table S3: The summarized statistics of 3D QSAR models for D4 subtype selectivity; Supplementary Figure S1: 2D structures of protonated piperazine derivatives used in this study; Supplementary Figure S2: Principal component analysis (PCA) of the training and test sets based on ECFP6 (A) and FCFP6 (B); Supplementary Figure S3: The 3D QSAR model for activity of D3 subtype with compound 84 (A and D), 106 (B and E), and 4 (C and F); Supplmentary Figure S4: The 3D QSAR model for activity of D4 subtype with compound 103 (A and C), and 5 (B and D); Supplementary Figure S5: Correlation plots between the experimental selectivity and predicted selectivity values of 3D QSAR models for D4/D2 (A) or D4/D3 (B) selectivity; Supplementary Figure S6: The contour maps of 3D QSAR models with D4-selective compounds.
