# Supplementary material for: Structural Basis for D3/D4-Selective Antagonism of Piperazinylalkyl Pyrazole/Isoxazole Analogs
Source: Molecules. 2025 Sep 28;30(19):3917. doi: 10.3390/molecules30193917 (PMC12526434; doi:10.3390/molecules30193917)
Supplement: Supplementary file 1 [file molecules-30-03917-s001.zip › molecules-3886214-supplementary.pdf]

Supplementary Information

Structural Basis for D3/D4-Selective Antagonism of Piperazinylalkyl Pyrazole/Isoxazole Analogs

Kwang-Eun Choi [1], Seong Hun Jang [2], Woo-Kyu Park [3], Kyoung Tai No [4], Hun Yeong Koh [5], Ae Nim Pae [6,*] and Nam-Chul Cho [1,*]

[1] Drug Information Platform Center, Korea Research Institute of Chemical Technology, Daejeon 34114, Republic of Korea
[2] R&D Center, PharmCADD, Busan 331, Republic of Korea
[3] Division of Drug Discovery Research, Korea Research Institute of Chemical Technology, Daejeon 34114, Republic of Korea
[4] Department of Integrative Biotechnology, Yonsei University, Seoul 120749, Republic of Korea
[5] Department of Chemistry, Inha University, Nam-gu, Incheon 402751, Republic of Korea
[6] Center of Brain Disorders, Brain Science Institute, Korea Institute of Science & Technology, Seoul 02792, Republic of Korea
* Correspondence: anpae@kist.re.kr (A.N.P.); nccho@krict.re.kr (N.-C.C.)

Supplementary Results

3D-QSAR models for D4 selectivity over D2 and D3 subtypes

To further clarify the molecular determinants of subtype selectivity, we generated 3D-QSAR models using the activity ratios $pIC_{50}(D4) - pIC_{50}(D2)$ and $pIC_{50}(D4) - pIC_{50}(D3)$ as dependent variables. The models were constructed with maximum common substructure–based molecular alignment (**Figure 2)** and validated by partial least squares (PLS) regression analysis. The D4/D2 selectivity model achieved $r^2 = 0.872$, $q^2 = 0.478$, and $Q^2 = 0.434$, whereas the D4/D3 model yielded $r^2 = 0.952$, $q^2 = 0.752$, and $Q^2 = 0.790$ (**Supplementary Table S3**). The strong correlation between experimental and predicted selectivity values is illustrated in **Supplementary Figure S5.**

Contour map analysis of the selectivity models (**Supplementary Figure S6**) revealed consistent steric and electrostatic features in the 4-phenylpiperazine moiety. Green and red contours at the meta- and para-positions indicated that bulky electronegative substituents at these sites are favorable for enhancing D4 selectivity. However, a large yellow contour also appeared near the same region, suggesting that steric tolerance is limited and overly bulky substituents may be detrimental. This trend is supported by experimental data: compounds **25**, **49**, **50**, **93**, and **103**, which bear excessively bulky groups such as CF_3, diphenylmethyl, or N-benzoimidazol-2-one, displayed reduced D4 selectivity compared with D2 or D3.

In contrast to the relatively conserved patterns at the 4-phenylpiperazine, the contour maps around the pyrazole/isoxazole moieties differed between the two models. For D4/D2 selectivity, a green steric contour at the N1-position of the pyrazole suggested that bulky substituents at this site enhance selectivity. This is consistent with the superior selectivity of N1-phenyl pyrazoles (compounds **29–38**) compared with N2-substituted analogs (compounds **16–28**). For D4/D3 selectivity, the favorable steric region shifted to the 5-position of the pyrazole. A red contour near this site indicated that electronegative substituents are beneficial, whereas a blue contour at a more distal region implied that electropositive groups may also contribute. The observed selectivity trends—n-propyl > i-propyl > methoxymethyl > hydrogen at the 5-position—are consistent with this prediction, exemplified by

compounds **47**, **22**, and **100**, which showed approximately 600-, 36-, and 6-fold selectivity for D4 over D3, respectively.

Collectively, these results demonstrate that D4 selectivity arises from a combination of steric and electrostatic effects. The 4-phenylpiperazine moiety confers a sterically constrained requirement for electronegative substituents at the meta-/para-positions, while the pyrazole moiety provides subtype-specific opportunities: N1-substitution enhances D4/D2 selectivity, whereas 5-substitution favors D4/D3 selectivity. These insights provide a structural rationale for observed SAR trends and offer practical guidelines for designing ligands with enhanced D4 preference.

Supplementary Table S1. The structure of compounds for training and test set

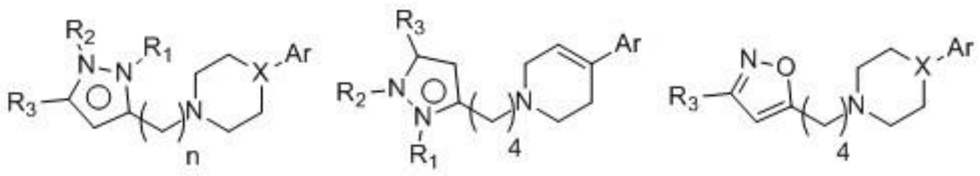

A B C

Compound	Core	n	X	R1	R2	R3	Ar	Set
1	A	3	N	-	*t*-butyl	Ph	Ph	T
2	A	3	N	-	*t*-butyl	Ph	*o*-flouro-Ph	T
3	A	3	N	-	*t*-butyl	Ph	*p*-chloro-ph	T
4	A	3	N	-	*t*-butyl	Ph	*o*, *p*-dimethyl-Ph	R
5	A	3	N	-	*t*-butyl	Ph	*m*, *p*-dimethyl-Ph	R
6	A	3	N	-	*t*-butyl	Ph	*o*, *m*-dimethyl-Ph	R
7	A	3	N	Ph	-	*n*-propyl	*m*, *p*-dimethyl-Ph	R
8	A	3	N	Ph	-	*n*-propyl	*o*, *m*-dimethyl-Ph	T
9	A	3	N	Ph	-	*n*-propyl	*m*, *p*-dichlro-Ph	T
10	A	3	N	Ph	-	ethyl	Ph	T
11	A	3	N	-	*t*-butyl	*n*-propyl	Ph	T
12	A	3	N	-	*t*-butyl	*n*-propyl	*o*-flouro-Ph	T
13	A	3	N	-	*t*-butyl	*n*-propyl	di-(*p*-fluoro-Ph)-CH	T
14	A	3	N	-	*t*-butyl	*n*-propyl	*o*, *p*-dimethyl-Ph	T
15	A	3	N	*p*-CF_3-Ph	-	*n*-propyl	*o*-fluoro-Ph	T
16	A	4	N	Ph	-	*i*-propyl	Ph	R
17	A	4	N	Ph	-	*i*-propyl	*o*-fluoro-Ph	R
18	A	4	N	Ph	-	*i*-propyl	*o*, *m*-dimethyl-Ph	T
19	A	4	N	Ph	-	*i*-propyl	*m*, *p*-dimethyl-Ph	R
20	A	4	N	Ph	-	*i*-propyl	*o*-methoxy-Ph	R
21	A	4	N	Ph	-	*i*-propyl	*p*-fluoro-Ph	R
22	A	4	N	Ph	-	*i*-propyl	*p*-chloro-Ph	R
23	B	-	-	Ph	-	*i*-propyl	*p*-chloro-Ph	R
24	A	4	C	Ph	-	*i*-propyl	N-benzoimidazole-2-one	T
25	A	4	N	Ph	-	*i*-propyl	*m*-CF_3-Ph	R
26	A	4	N	Ph	-	*i*-propyl	2-pyrimidine	R
27	A	4	NH	Ph	-	*i*-propyl	di-(*p*-fluro-Ph)-CH	T
28	A	4	N	Ph	-	*i*-propyl	2-pyridine	R
29	A	4	N	-	Ph	*i*-propyl	Ph	T
30	A	4	N	-	Ph	*i*-propyl	*o*-fluoro-Ph	R
31	A	4	N	-	Ph	*i*-propyl	*m*, *p*-dimethy-Ph	R
32	A	4	N	-	Ph	*i*-propyl	*o*-methoxy-Ph	T
33	A	4	N	-	Ph	*i*-propyl	*p*-fluoro-Ph	R
34	A	4	N	-	Ph	*i*-propyl	*p*-chloro-Ph	R
35	B	-		-	Ph	*i*-propyl	*p*-chloro-Ph	T
36	A	4	C	-	Ph	*i*-propyl	N-benzoimidazole-2-one	R
37	A	4	N	-	Ph	*i*-propyl	*m*-CF_3-Ph	R
38	A	4	N	-	Ph	*i*-propyl	2-pyrimidine	T
39	A	4	NH	-	Ph	*i*-propyl	di-(*p*-fluro-Ph)-CH	R

Supplementary Table S1. The structure of compounds for training and test set (continued)

40	A	4	N	-	Ph	*i*-propyl	2-pyridine	T
41	A	4	N	Ph	-	*n*-propyl	Ph	R
42	A	4	N	Ph	-	*n*-propyl	*o*-fluoro-Ph	R
43	A	4	N	Ph	-	*n*-propyl	*o*, *m*-dimethyl-Ph	R
44	A	4	N	Ph	-	*n*-propyl	*m*, *p*-dimethyl-Ph	R
45	A	4	N	Ph	-	*n*-propyl	*o*-methoxy-Ph	T
46	A	4	N	Ph	-	*n*-propyl	*p*-fluoro-Ph	R
47	A	4	N	Ph	-	*n*-propyl	*p*-chloro-Ph	R
48	B	-	-	Ph	-	*n*-propyl	*p*-chloro-Ph	R
49	A	4	C	Ph	-	*n*-propyl	N-benzoimidazole-2-one	R
50	A	4	N	Ph	-	*n*-propyl	2-pyrimidine	R
51	A	4	NH	Ph	-	*n*-propyl	di-(*p*-fluro-Ph)-CH	T
52	A	4	N	Ph	-	*n*-propyl	2-pyridine	R
53	A	4	N	-	Ph	*n*-propyl	Ph	T
54	A	4	N	-	Ph	*n*-propyl	*o*-fluoro-Ph	R
55	A	4	N	-	Ph	*n*-propyl	*o*, *m*-dimethyl-Ph	R
56	A	4	N	-	Ph	*n*-propyl	*m*, *p*-dimethyl-Ph	T
57	A	4	N	-	Ph	*n*-propyl	*o*-methoxy-Ph	R
58	A	4	N	-	Ph	*n*-propyl	*p*-fluoro-Ph	R
59	A	4	N	-	Ph	*n*-propyl	*p*-chloro-Ph	T
60	B	-	-	-	Ph	*n*-propyl	*p*-chloro-Ph	T
61	A	4	C	-	Ph	*n*-propyl	N-benzoimidazole-2-one	T
62	A	4	N	-	Ph	*n*-propyl	*m*-CF_3-Ph	T
63	A	4	N	-	Ph	*n*-propyl	2-pyrimidine	T
64	A	4	NH	-	Ph	*n*-propyl	di-(*p*-fluoro-Ph)-CH	R
65	A	4	N	-	Ph	*n*-propyl	2-pyridine	T
66	A	3	N	Ph		*n*-propyl	*o*-fluoro-Ph	R
67	A	3	N	*m*-chloro-Ph	-	*n*-propyl	*o*-fluoro-Ph	R
68	A	3	N	*p*-methoxy-Ph	-	*n*-propyl	*o*-fluoro-Ph	R
69	A	3	N	*p*-methyl-Ph	-	*n*-propyl	*o*-fluoro-Ph	R
70	A	3	N	*p*-fluoro-Ph	-	*n*-propyl	*o*-fluoro-Ph	T
71	A	3	N	*p*-chloro-Ph	-	*n*-propyl	*o*-fluoro-Ph	T
72	A	3	N	*p*-OCF_3-Ph	-	*n*-propyl	*o*-fluoro-Ph	T
73	A	3	N	*p*-nitro-Ph	-	*n*-propyl	*o*-fluoro-Ph	T
74	A	3	N	BZ	-	*n*-propyl	*o*-fluoro-Ph	T
75	A	3	N	*m*-nitro-Ph	-	*n*-propyl	*o*-fluoro-Ph	R
76	A	3	N	2-pyridine	-	*n*-propyl	*o*-fluoro-Ph	R
77	A	3	N	Ph	-	*n*-butyl	*o*-fluoro-Ph	R
78	A	3	N	BZ	-	*n*-butyl	*o*-fluoro-Ph	T
79	A	3	N	*o*-chloro-Ph	-	*n*-butyl	*o*-fluoro-Ph	T
80	A	3	N	*p*-methyl-Ph	-	*n*-butyl	*o*-fluoro-Ph	R
81	A	4	N	-	Ph	methylmethoxy	Ph	R
82	A	4	N	-	Ph	methylmethoxy	*o*-fluoro-Ph	R
83	A	4	N	-	Ph	methylmethoxy	*p*-CF_3-Ph	R
84	A	4	N	-	Ph	methylmethoxy	*o*, *m*-dimethyl-Ph	R
85	A	4	N	-	Ph	methylmethoxy	2-pyrimidine	T
86	A	4	N	-	Ph	methylmethoxy	di-(*p*-fluoro-Ph)-CH	R

Supplementary Table S1. The structure of compounds for training and test set (continued)

87	A	4	N	-	Ph	methylmethoxy	*m*, *p*-dimethyl-Ph	R
88	A	4	N	-	Ph	methylmethoxy	*o*-methoxy-Ph	T
89	A	4	N	-	Ph	methylmethoxy	*p*-fluoro-Ph	R
90	A	4	N	-	Ph	methylmethoxy	*p*-chloro-Ph	T
91	A	4	N	Ph	-	methylmethoxy	Ph	T
92	A	4	N	Ph	-	methylmethoxy	*o*-fluoro-Ph	R
93	A	4	NH	Ph	-	methylmethoxy	*m*-CF_3-Ph	T
94	A	4	N	Ph	-	methylmethoxy	*o*, *m*-dimethyl-Ph	R
95	A	4	N	Ph	-	methylmethoxy	2-pyrimidine	R
96	A	4	NH^+	Ph	-	methylmethoxy	di-(*p*-fluoro-Ph)-CH	R
97	A	4	N	Ph	-	methylmethoxy	*m*, *p*-dimethyl-Ph	R
98	A	4	N	Ph	-	methylmethoxy	*o*-methoxy-Ph	R
99	A	4	N	Ph	-	methylmethoxy	*p*-fluoro-Ph	T
100	A	4	N	Ph	-	methylmethoxy	*p*-chloro-Ph	R
101	A	3	N	-	Ph	methylmethoxy	*o*, *m*-dimethyl-Ph	T
102	A	3	N	-	Ph	methylmethoxy	*p*-chloro-Ph	T
103	A	3	NH	-	Ph	methylmethoxy	di-(*p*-fluoro-Ph)-CH	R
104	A	3	N	-	Ph	methylmethoxy	*p*-chloro-Ph	R
105	A	3	N	-	Ph	methylmethoxy	*p*-CF_3-Ph	T
106	A	3	N	Ph	-	methylmethoxy	*o*, *m*-dimethyl-Ph	R
107	A	3	N	Ph	-	methylmethoxy	*p*-chloro-Ph	R
108	A	3	N	Ph	-	methylmethoxy	*o*-methoxy-Ph	T
109	A	3	N	Ph	-	methylmethoxy	di-(*p*-fluoro-Ph)-CH	T
110	A	3	N	Ph	-	methylmethoxy	*p*-fluoro-Ph	T
111	A	3	N	Ph	-	methylmethoxy	*m*-CF_3-Ph	T
112	C	-	N	-	-	4-F-Ph	Ph	R
113	C	-	N	-	-	3-nitro-Ph	Ph	T
114	C	-	N	-	-	3, 4-dimethoxy-Ph	Ph	T
115	C	-	N	-	-	2, 4-dimethoxy-Ph	Ph	R
116	C	-	N	-	-	2-thiophene	Ph	T
117	C	-	N	-	-	Ph	Ph	T
118	C	-	N	-	-	*(E)*-styryl	Ph	T
119	C	-	N	-	-	3, 4-dimethoxy-Ph	*o*-methyl-Ph	T
120	C	-	N	-	-	2-thiophene	*o*-methyl-Ph	T
121	C	-	N	-	-	Ph	*o*-methyl-Ph	R
122	C	-	N	-	-	4-fluoro-Ph	*o*-methoxy-Ph	T
123	C	-	N	-	-	3-nitro-Ph	*o*-methoxy-Ph	T
124	C	-	N	-	-	3, 4-dimethoxy-Ph	*o*-methoxy-Ph	R
125	C	-	N	-	-	2, 4-dimethoxy-Ph	*o*-methoxy-Ph	T
126	C	-	N	-	-	2-thiophene	*o*-methoxy-Ph	T
127	C	-	N	-	-	Ph	*o*-methoxy-Ph	T
128	C	-	N	-	-	*(E)*-styryl	*o*-methoxy-Ph	T
129	C	-	N	-	-	3-nitro-Ph	*o*-ethoxy-Ph	T
130	C	-	N	-	-	3, 4-methoxy-Ph	*o*-ethoxy-Ph	T
131	C	-	N	-	-	2-thiophene	*o*-ethoxy-Ph	T
132	C	-	N	-	-	Ph	*o*-ethoxy-Ph	R
133	C	-	N	-	-	3, 4-methoxy-Ph	*o*-fluoro-Ph	R

Supplementary Table S1. The structure of compounds for training and test set (continued)

134	C	-	N	-	-	*(E)*-styryl	o-fluoro-Ph	T
135	C	-	NH	-	-	4-fluro-Ph	di-Ph-CH	R
136	C	-	NH	-	-	3-nitro-Ph	di-Ph-CH	T
137	C	-	NH	-	-	3, 4-dimethoxy-Ph	di-Ph-CH	T
138	C	-	NH	-	-	2, 3-dimethoxy-Ph	di-Ph-CH	R
139	C	-	NH	-	-	2-thiophene	di-Ph-CH	T
140	C	-	NH	-	-	Ph	di-Ph-CH	T
141	C	-	NH	-	-	*(E)*-styryl	di-Ph-CH	T

Ph: phenyl, BZ: benzenyl, R: Training set, T: Test set.

Supplementary Table S2. The experimental values and predicted values of 3D QSAR models.

Name	SET	D2			D3			D4			D4/D2			D4/D3		
		Exp	Pred	Residual	Exp	Pred	Residual	Exp	Pred	Residual	ES	PS	Residual	ES	PS	Residual
1	T	5.810	6.173	-0.363	5.000	5.577	-0.577	8.066	7.974	0.092	2.256	2.116	0.140	3.066	2.358	0.708
2	T	5.976	6.299	-0.323	5.379	5.603	-0.224	8.022	7.877	0.146	2.047	1.712	0.335	2.643	2.233	0.410
3	T	6.158	5.991	0.167	5.000	5.114	-0.114	7.975	8.343	-0.369	1.817	2.592	-0.775	2.975	3.156	-0.181
4	R	5.838	5.818	0.020	5.000	5.073	-0.073	7.939	7.808	0.131	2.101	2.116	-0.015	2.939	2.806	0.134
5	R	6.351	6.295	0.055	6.169	5.672	0.496	8.174	8.398	-0.224	1.823	2.289	-0.466	2.005	2.737	-0.732
6	R	6.425	6.463	-0.038	5.591	5.758	-0.166	8.357	7.932	0.425	1.932	1.804	0.128	2.765	2.148	0.617
7	R	6.336	6.271	0.065	5.279	5.558	-0.279	8.229	8.373	-0.144	1.893	2.128	-0.235	2.950	2.916	0.034
8	T	7.060	6.890	0.170	6.157	6.087	0.070	8.456	7.490	0.966	1.395	0.840	0.556	2.299	1.319	0.979
9	T	6.883	6.398	0.484	5.689	5.478	0.211	8.886	8.043	0.843	2.003	1.801	0.203	3.197	2.549	0.648
10	T	6.377	5.614	0.762	5.976	5.494	0.482	8.114	6.743	1.371	1.737	1.154	0.583	2.137	1.257	0.881
11	T	5.954	6.040	-0.086	5.773	5.898	-0.124	7.347	7.386	-0.039	1.393	1.556	-0.163	1.573	1.429	0.145
12	T	5.953	6.180	-0.227	6.485	5.948	0.537	7.357	7.312	0.044	1.403	1.171	0.233	0.871	1.300	-0.429
13	T	6.640	6.172	0.468	5.421	5.789	-0.368	8.538	6.357	2.181	1.897	0.385	1.512	3.117	0.383	2.733
14	T	6.032	5.677	0.356	6.573	5.434	1.139	7.276	7.271	0.004	1.243	1.600	-0.357	0.702	1.885	-1.183
15	T	5.972	6.512	-0.540	6.277	5.989	0.288	7.060	7.340	-0.280	1.089	0.844	0.244	0.783	1.283	-0.500
16	R	6.235	6.332	-0.097	6.660	6.520	0.141	6.928	7.022	-0.095	0.692	0.661	0.031	0.267	0.402	-0.135
17	R	6.650	6.614	0.035	6.733	6.566	0.167	6.990	6.929	0.062	0.340	0.388	-0.048	0.257	0.275	-0.018
18	T	6.446	6.750	-0.304	6.700	6.566	0.133	7.124	7.204	-0.081	0.678	0.499	0.180	0.424	0.549	-0.125
19	R	5.988	6.035	-0.048	5.971	6.357	-0.386	7.530	7.603	-0.073	1.543	1.339	0.203	1.560	1.259	0.301
20	R	6.881	6.954	-0.073	6.303	6.477	-0.174	7.471	7.308	0.163	0.590	0.493	0.097	1.168	0.730	0.438
21	R	5.723	5.590	0.133	5.745	5.682	0.063	7.517	7.285	0.232	1.794	1.649	0.145	1.772	1.516	0.256
22	R	5.554	5.590	-0.036	6.048	5.776	0.273	7.606	7.693	-0.087	2.051	1.932	0.120	1.557	1.787	-0.230
23	R	5.796	5.654	0.143	6.558	6.203	0.356	6.774	6.842	-0.068	0.978	0.986	-0.008	0.216	0.610	-0.394
24	T	6.075	6.229	-0.154	5.928	5.922	0.006	6.350	6.531	-0.182	0.275	0.196	0.078	0.422	0.611	-0.189
25	R	6.232	6.118	0.114	6.085	6.360	-0.276	6.383	6.655	-0.272	0.152	0.348	-0.196	0.298	0.297	0.002
26	R	5.292	5.242	0.050	5.083	5.455	-0.372	6.848	6.732	0.116	1.556	1.164	0.392	1.765	1.313	0.452
27	T	5.925	5.833	0.093	5.359	5.776	-0.417	6.407	6.348	0.059	0.482	0.516	-0.034	1.048	0.338	0.710
28	R	5.385	5.434	-0.049	5.694	5.707	-0.013	6.687	6.743	-0.056	1.302	1.101	0.202	0.993	0.876	0.117

Supplementary Table S2. The experimental and predicted values of compounds on 3D QSAR models (continued)

29	T	6.000	5.795	0.205	6.523	6.120	0.403	6.627	7.022	-0.395	0.627	1.174	-0.547	0.104	0.905	-0.801
30	R	6.027	6.288	-0.262	6.659	6.488	0.171	6.875	7.025	-0.150	0.848	0.536	0.312	0.216	0.473	-0.257
31	R	6.525	6.347	0.179	6.402	6.626	-0.223	7.752	7.586	0.166	1.227	1.138	0.089	1.350	0.954	0.396
32	T	7.693	6.253	1.440	6.987	6.426	0.561	7.257	6.952	0.305	-0.435	0.672	-1.107	0.270	0.451	-0.181
33	R	6.234	5.998	0.236	5.996	5.956	0.039	7.027	7.219	-0.191	0.793	1.316	-0.523	1.032	1.182	-0.150
34	R	5.903	5.992	-0.089	6.104	5.999	0.105	7.703	7.660	0.043	1.800	1.635	0.165	1.599	1.540	0.060
35	T	6.121	6.018	0.104	6.362	6.362	-0.001	6.732	6.867	-0.135	0.610	0.754	-0.143	0.370	0.480	-0.110
36	R	6.448	6.420	0.028	6.109	6.167	-0.058	6.401	6.340	0.060	-0.047	-0.125	0.078	0.292	0.145	0.147
37	R	6.399	6.361	0.038	6.808	6.645	0.163	6.851	6.620	0.231	0.452	0.131	0.321	0.043	-0.053	0.096
38	T	5.470	5.536	-0.066	5.619	5.834	-0.216	6.411	6.610	-0.199	0.941	0.856	0.085	0.793	0.791	0.002
39	R	6.085	6.129	-0.044	6.820	6.600	0.220	5.990	5.965	0.025	-0.095	-0.131	0.036	-0.831	-0.779	-0.052
40	T	5.565	5.536	0.029	6.026	5.820	0.207	6.337	5.961	0.376	0.773	0.553	0.219	0.311	0.056	0.256
41	R	6.276	6.150	0.126	6.158	6.107	0.052	7.374	7.411	-0.037	1.098	1.340	-0.243	1.215	1.381	-0.166
42	R	6.834	6.663	0.172	6.643	6.570	0.073	6.871	7.025	-0.155	0.036	0.397	-0.360	0.228	0.488	-0.260
43	R	6.717	6.898	-0.181	6.973	6.975	-0.001	6.973	7.237	-0.263	0.256	0.548	-0.292	0.000	0.289	-0.289
44	R	6.240	6.208	0.032	6.407	6.317	0.089	8.187	8.367	-0.180	1.947	2.049	-0.101	1.780	2.206	-0.426
45	T	6.924	6.689	0.236	6.383	6.587	-0.205	7.400	7.264	0.136	0.476	0.755	-0.279	1.017	0.670	0.348
46	R	5.546	5.761	-0.216	5.834	5.764	0.070	8.013	7.752	0.261	2.467	1.917	0.551	2.179	2.037	0.142
47	R	6.179	6.061	0.119	5.375	5.691	-0.315	8.195	8.082	0.113	2.016	2.079	-0.063	2.820	2.435	0.385
48	R	6.205	6.189	0.016	5.854	6.311	-0.456	7.403	7.170	0.234	1.199	0.959	0.239	1.549	0.907	0.642
49	R	6.571	6.683	-0.112	6.152	6.257	-0.105	6.584	6.536	0.048	0.013	-0.075	0.088	0.431	0.337	0.095
50	R	5.922	5.894	0.028	5.754	5.806	-0.052	6.987	6.982	0.005	1.065	1.012	0.053	1.232	1.273	-0.040
51	T	6.745	6.054	0.691	6.967	6.294	0.674	6.473	6.224	0.248	-0.272	0.347	-0.619	-0.495	-0.304	-0.191
52	R	5.843	5.877	-0.034	5.941	5.623	0.318	6.815	6.759	0.055	0.972	1.099	-0.127	0.874	1.065	-0.191
53	T	6.058	5.948	0.109	6.406	6.632	-0.226	7.074	7.361	-0.287	1.016	1.312	-0.295	0.668	0.827	-0.160
54	R	6.353	6.240	0.113	6.323	6.512	-0.189	6.811	6.657	0.154	0.458	0.407	0.051	0.488	0.274	0.215
55	R	6.215	6.272	-0.057	7.015	6.901	0.114	7.313	7.319	-0.005	1.098	0.931	0.168	0.298	0.511	-0.212
56	T	6.097	6.109	-0.012	6.238	6.774	-0.536	7.939	7.813	0.126	1.842	1.463	0.380	1.701	1.179	0.522
57	R	7.061	7.000	0.061	6.849	6.816	0.032	7.509	7.484	0.024	0.448	0.562	-0.114	0.660	0.697	-0.037
58	R	5.504	5.690	-0.186	6.079	6.119	-0.040	7.376	7.389	-0.014	1.872	1.625	0.247	1.297	1.341	-0.044

Supplementary Table S2. The experimental and predicted values of compounds on 3D QSAR models (continued)

59	T	5.687	5.957	-0.270	6.302	6.077	0.226	7.790	7.340	0.450	2.103	1.531	0.572	1.488	1.323	0.165
60	T	5.599	5.778	-0.179	6.464	6.516	-0.052	6.973	7.035	-0.062	1.374	1.045	0.328	0.509	0.620	-0.111
61	T	6.180	6.242	-0.063	5.891	6.323	-0.432	5.611	6.449	-0.838	-0.569	0.143	-0.712	-0.280	0.242	-0.523
62	T	6.122	6.089	0.033	6.795	6.735	0.060	6.052	6.862	-0.811	-0.070	0.476	-0.546	-0.743	0.250	-0.992
63	T	5.872	5.227	0.645	5.753	5.997	-0.244	6.519	6.753	-0.234	0.648	1.183	-0.536	0.767	0.940	-0.173
64	R	6.418	6.318	0.100	5.719	6.132	-0.413	5.991	6.248	-0.257	-0.428	0.119	-0.546	0.271	0.103	0.169
65	T	5.965	5.576	0.389	6.204	5.888	0.316	6.878	6.504	0.374	0.913	0.972	-0.059	0.675	0.699	-0.024
66	R	6.618	6.532	0.085	5.850	5.803	0.048	7.130	7.427	-0.296	0.513	0.988	-0.476	1.280	1.550	-0.271
67	R	6.624	6.522	0.102	5.871	5.890	-0.020	7.413	7.409	0.005	0.790	0.990	-0.201	1.543	1.434	0.109
68	R	5.930	6.120	-0.190	5.910	5.933	-0.023	7.255	7.441	-0.186	1.325	1.172	0.153	1.344	1.447	-0.102
69	R	6.741	6.617	0.125	6.373	5.967	0.406	7.465	7.526	-0.061	0.723	0.993	-0.269	1.091	1.488	-0.397
70	T	6.442	6.557	-0.114	6.037	5.978	0.059	7.278	7.597	-0.319	0.836	1.096	-0.260	1.241	1.544	-0.302
71	T	6.673	6.507	0.166	5.924	6.034	-0.111	7.010	7.557	-0.547	0.337	1.088	-0.751	1.086	1.435	-0.349
72	T	6.731	6.513	0.219	6.074	5.982	0.091	7.650	7.593	0.057	0.918	1.087	-0.169	1.576	1.539	0.037
73	T	5.734	6.049	-0.315	5.856	6.061	-0.205	7.379	7.293	0.086	1.645	1.148	0.497	1.522	1.128	0.394
74	T	6.285	6.359	-0.074	6.092	5.876	0.215	7.110	7.378	-0.268	0.825	1.049	-0.224	1.018	1.437	-0.419
75	R	5.752	5.752	-0.001	6.026	5.960	0.066	7.313	7.246	0.067	1.562	1.285	0.276	1.288	1.177	0.111
76	R	6.158	6.219	-0.061	6.023	5.879	0.144	7.857	7.585	0.272	1.699	1.316	0.383	1.834	1.589	0.245
77	R	6.205	6.302	-0.098	5.587	5.730	-0.143	7.780	7.672	0.108	1.575	1.153	0.423	2.193	1.896	0.297
78	T	6.055	6.315	-0.260	5.000	5.816	-0.816	6.942	7.242	-0.300	0.886	0.874	0.012	1.942	1.396	0.545
79	T	6.025	6.384	-0.358	6.057	5.770	0.286	7.456	7.589	-0.133	1.430	1.069	0.362	1.399	1.776	-0.377
80	R	6.548	6.511	0.036	5.908	5.875	0.033	7.282	7.422	-0.139	0.734	0.788	-0.054	1.374	1.509	-0.135
81	R	5.112	5.227	-0.116	6.343	6.419	-0.076	6.294	6.380	-0.086	1.183	1.245	-0.063	-0.049	0.109	-0.158
82	R	5.729	5.746	-0.017	6.796	6.766	0.030	6.189	6.229	-0.039	0.460	0.526	-0.066	-0.607	-0.426	-0.181
83	R	5.933	5.782	0.151	6.599	6.734	-0.135	6.395	6.419	-0.024	0.462	0.816	-0.354	-0.204	-0.092	-0.111
84	R	6.077	6.112	-0.035	7.071	7.113	-0.042	6.390	6.478	-0.088	0.313	0.613	-0.300	-0.680	-0.541	-0.139
85	T	5.000	5.360	-0.360	5.427	6.236	-0.809	5.733	6.315	-0.582	0.733	1.015	-0.282	0.305	0.079	0.226
86	R	5.660	5.842	-0.182	6.752	6.508	0.244	6.040	6.044	-0.004	0.381	0.265	0.116	-0.712	-0.619	-0.093
87	R	5.164	5.212	-0.048	6.377	6.577	-0.201	7.456	7.320	0.136	2.292	1.988	0.304	1.079	0.970	0.109
88	T	5.942	5.849	0.093	7.081	6.828	0.253	6.278	6.337	-0.059	0.336	0.657	-0.321	-0.803	-0.432	-0.370

Supplementary Table S2. The experimental and predicted values of compounds on 3D QSAR models (continued)

89	R	5.000	4.918	0.082	5.821	5.997	-0.176	6.395	6.702	-0.307	1.395	1.805	-0.410	0.574	0.855	-0.281
90	T	5.000	5.181	-0.181	5.957	5.962	-0.006	7.347	7.018	0.329	2.347	1.924	0.423	1.390	1.178	0.212
91	T	5.495	5.137	0.358	6.186	6.046	0.140	5.795	6.328	-0.533	0.300	1.125	-0.825	-0.391	0.343	-0.734
92	R	5.431	5.562	-0.132	6.484	6.474	0.011	6.056	6.101	-0.045	0.625	0.356	0.269	-0.428	-0.381	-0.047
93	T	5.976	5.675	0.301	6.275	6.417	-0.142	5.989	6.413	-0.424	0.013	0.735	-0.723	-0.286	0.105	-0.391
94	R	6.151	5.998	0.152	6.807	6.897	-0.090	6.402	6.266	0.137	0.252	0.362	-0.110	-0.405	-0.633	0.228
95	R	5.000	5.073	-0.073	5.547	5.622	-0.075	5.597	5.750	-0.153	0.597	0.763	-0.166	0.049	0.069	-0.020
96	R	6.045	6.148	-0.103	7.036	6.604	0.433	6.167	6.027	0.140	0.122	0.152	-0.030	-0.869	-0.894	0.025
97	R	5.256	5.136	0.120	5.974	6.225	-0.251	6.889	7.243	-0.354	1.634	1.843	-0.210	0.915	1.146	-0.231
98	R	5.815	5.762	0.054	6.629	6.522	0.107	6.648	6.429	0.219	0.833	0.686	0.147	0.019	-0.155	0.174
99	T	5.000	4.743	0.257	5.685	5.736	-0.051	6.167	6.587	-0.420	1.167	1.650	-0.484	0.482	0.895	-0.413
100	R	5.000	5.052	-0.052	5.748	5.666	0.082	6.587	6.919	-0.332	1.587	1.793	-0.206	0.839	1.273	-0.434
101	T	5.000	5.493	-0.493	6.162	6.001	0.161	5.891	5.571	0.320	0.891	0.244	0.647	-0.271	-0.468	0.198
102	T	5.000	5.024	-0.024	5.357	5.362	-0.005	6.240	6.049	0.191	1.240	1.090	0.149	0.883	0.604	0.279
103	R	5.485	5.518	-0.033	6.096	5.794	0.302	5.301	5.299	0.002	-0.184	-0.104	-0.080	-0.795	-0.736	-0.059
104	R	5.000	5.008	-0.008	5.400	5.293	0.107	5.488	5.532	-0.044	0.488	0.737	-0.249	0.088	0.175	-0.087
105	T	5.000	5.330	-0.330	5.904	5.979	-0.075	5.744	5.091	0.653	0.744	-0.256	1.000	-0.161	-0.898	0.737
106	R	5.242	5.260	-0.018	6.539	6.444	0.095	5.782	5.578	0.204	0.540	0.223	0.318	-0.757	-0.999	0.242
107	R	5.000	4.803	0.197	5.513	5.395	0.118	6.269	6.233	0.036	1.269	1.279	-0.010	0.756	0.749	0.007
108	T	5.613	5.108	0.504	6.099	6.137	-0.039	6.137	5.564	0.573	0.525	0.334	0.191	0.039	-0.724	0.763
109	T	5.603	5.514	0.089	7.367	6.094	1.273	5.444	5.220	0.224	-0.158	-0.241	0.082	-1.922	-1.104	-0.818
110	T	5.000	4.518	0.482	5.204	5.355	-0.150	5.759	5.924	-0.166	0.759	1.175	-0.416	0.554	0.486	0.069
111	R	5.044	5.207	-0.163	5.896	6.082	-0.185	5.630	5.567	0.063	0.585	0.239	0.346	-0.267	-0.514	0.248
112	R	5.597	5.629	-0.032	7.921	7.780	0.141	7.018	6.894	0.123	1.421	1.340	0.081	-0.903	-0.816	-0.087
113	T	5.833	5.631	0.202	7.222	7.641	-0.419	5.770	6.696	-0.926	-0.062	1.105	-1.167	-1.452	-0.822	-0.630
114	T	5.470	5.625	-0.155	7.620	7.969	-0.349	6.111	6.680	-0.569	0.641	1.123	-0.482	-1.509	-1.102	-0.407
115	R	5.658	5.689	-0.031	7.796	7.837	-0.041	7.119	6.968	0.151	1.461	1.508	-0.047	-0.677	-0.829	0.153
116	T	5.383	5.586	-0.202	7.824	7.075	0.749	6.932	6.677	0.255	1.548	1.154	0.394	-0.892	-0.355	-0.537
117	T	5.473	5.537	-0.064	7.745	7.381	0.364	6.267	6.745	-0.478	0.794	1.263	-0.469	-1.478	-0.496	-0.982
118	T	5.012	5.584	-0.572	7.569	7.636	-0.067	6.461	6.794	-0.333	1.449	1.298	0.151	-1.108	-0.745	-0.362

Supplementary Table S2. The experimental and predicted values of compounds on 3D QSAR models (continued)

119	T	5.854	5.326	0.528	7.420	7.618	-0.198	6.384	6.342	0.042	0.530	0.861	-0.330	-1.036	-0.952	-0.084
120	T	5.676	5.227	0.449	6.788	6.652	0.135	6.333	6.314	0.019	0.656	0.886	-0.229	-0.455	-0.142	-0.313
121	R	5.186	5.170	0.016	6.535	7.020	-0.485	6.166	6.399	-0.233	0.979	1.018	-0.039	-0.369	-0.341	-0.028
122	T	6.494	6.368	0.126	7.638	8.138	-0.500	7.481	6.709	0.773	0.988	0.496	0.491	-0.157	-1.320	1.163
123	T	6.530	6.372	0.159	8.222	8.043	0.179	7.301	6.663	0.638	0.771	0.389	0.382	-0.921	-1.221	0.300
124	R	6.379	6.473	-0.094	8.284	8.389	-0.105	6.590	6.614	-0.024	0.211	0.312	-0.101	-1.694	-1.562	-0.132
125	T	6.357	5.647	0.710	8.187	7.632	0.555	7.469	6.739	0.730	1.112	1.156	-0.044	-0.719	-0.842	0.124
126	T	6.139	6.428	-0.289	8.328	7.477	0.851	7.678	6.617	1.061	1.539	0.323	1.216	-0.650	-0.776	0.125
127	T	5.763	5.465	0.298	8.022	7.262	0.761	6.703	6.383	0.320	0.941	0.790	0.150	-1.319	-0.682	-0.637
128	T	6.016	6.473	-0.458	7.824	8.093	-0.269	7.056	6.745	0.310	1.040	0.456	0.584	-0.768	-1.214	0.445
129	T	7.022	5.661	1.362	7.721	7.560	0.161	7.292	6.393	0.899	0.270	0.627	-0.357	-0.429	-0.978	0.549
130	T	6.924	6.215	0.709	8.585	8.103	0.482	7.553	7.034	0.519	0.628	0.900	-0.272	-1.032	-0.941	-0.091
131	T	6.712	6.489	0.223	8.409	7.396	1.013	7.921	6.976	0.945	1.209	0.615	0.593	-0.488	-0.380	-0.108
132	R	6.467	6.488	-0.021	8.046	7.780	0.266	7.469	7.069	0.399	1.001	0.722	0.279	-0.577	-0.587	0.010
133	R	5.288	5.160	0.128	7.699	7.716	-0.017	6.633	6.498	0.135	1.344	1.133	0.212	-1.066	-1.062	-0.004
134	T	6.233	5.496	0.737	8.000	7.699	0.301	7.237	6.559	0.678	1.004	0.947	0.057	-0.763	-1.034	0.271
135	R	5.835	5.839	-0.004	7.523	7.536	-0.013	5.871	5.904	-0.033	0.036	0.212	-0.177	-1.652	-1.667	0.015
136	T	5.818	5.840	-0.022	7.721	7.496	0.226	5.636	6.026	-0.391	-0.182	0.284	-0.466	-2.086	-1.477	-0.608
137	T	5.582	5.784	-0.202	7.699	7.692	0.007	5.613	5.809	-0.196	0.031	0.169	-0.138	-2.086	-1.862	-0.224
138	R	5.417	5.498	-0.081	7.796	7.701	0.095	5.952	5.975	-0.024	0.535	0.602	-0.067	-1.844	-1.808	-0.036
139	T	6.208	5.813	0.395	7.284	6.910	0.374	5.669	6.228	-0.560	-0.539	0.465	-1.004	-1.615	-0.768	-0.847
140	T	6.313	5.765	0.548	7.824	7.270	0.554	5.649	6.021	-0.372	-0.664	0.353	-1.017	-2.175	-1.248	-0.927
141	T	6.309	5.795	0.514	7.398	7.489	-0.091	6.220	6.039	0.181	-0.089	0.349	-0.438	-1.178	-1.469	0.291

R: Training set; T: Test set; Exp is the experimental pIC_{50}; Pred is the predicted pIC_{50}; ES is the estimated selectivity; PS is the predicted selectivity.

Supplementary Table S3. The summarized statistics of 3D QSAR models for D4 subtype selectivity.

PLS statistics	D4/D2	D4/D3
r^2	0.872	0.952
r^2 RMS residual error	0.2421	0.2447
q^2	0.478	0.752
q^2 RMS residual error	0.492	0.557
Number of component	3	4
Q^2	0.434	0.790
Q^2 RMS error	0.532	0.608
Mean absolute error	0.430	0.458

r^2 is the non-cross-validated regression coefficient; q^2 is the five-fold cross-validated regression correlation coefficient; Q^2 is the cross-validated correlation coefficient of the test set; RMS is the root mean square.

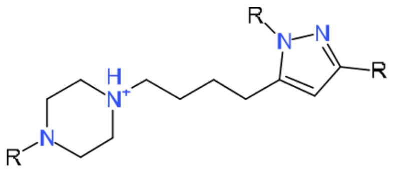

Piperazinylalkyl Pyrazole

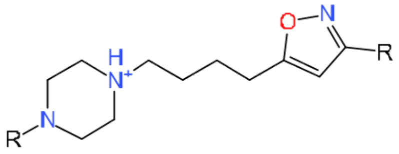

Piperazinylalkyl Isoxazole

Supplementary Figure S1. 2D structures of protonated piperazine derivatives used in this study.

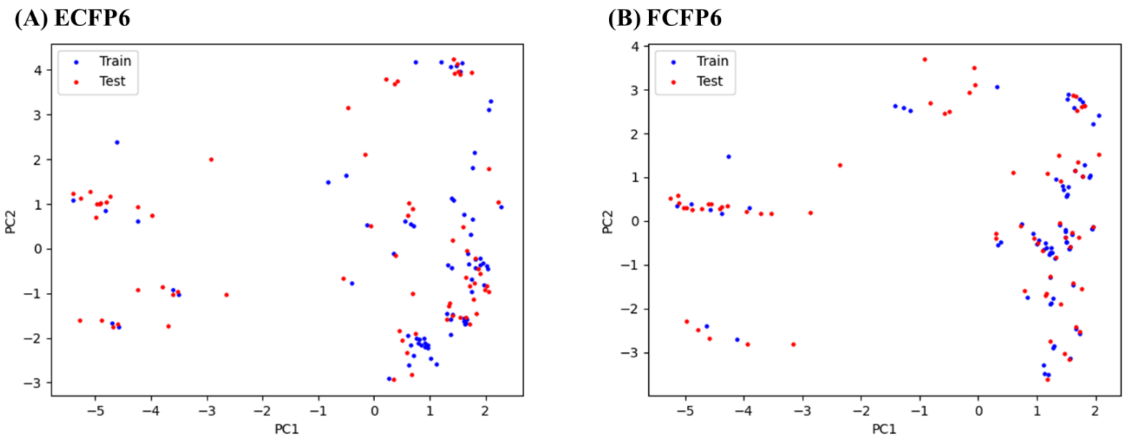

Supplementary Figure S2. Principal component analysis (PCA) of the training and test sets based on ECFP6 (A) and FCFP6 (B).

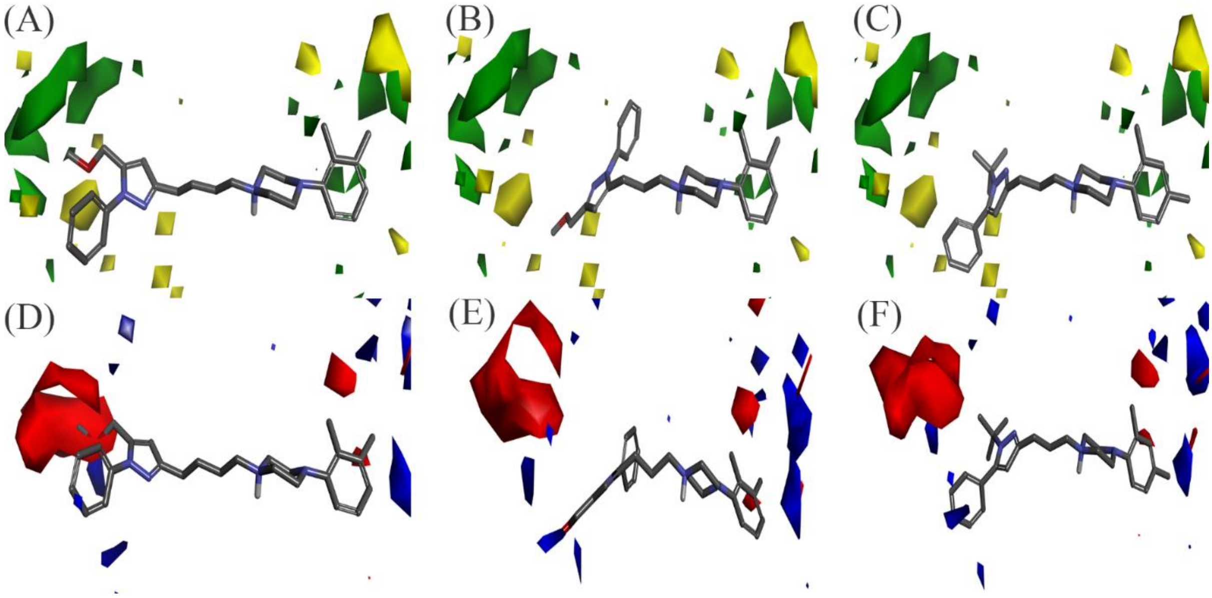

Supplementary Figure S3. The 3D QSAR model for activity of D3 subtype with compound 84 (A and D), 106 (B and E), and 4 (C and F). The green contour indicates regions where sterically bulky group increase an inhibitory activity, while the yellow contours indicate regions where sterically bulky groups decrease an inhibitory activity. The blue contour indicates regions where electropositive charged groups increase an inhibitory activity, while the red contour represent where electronegative charged groups improve an inhibitory activity.

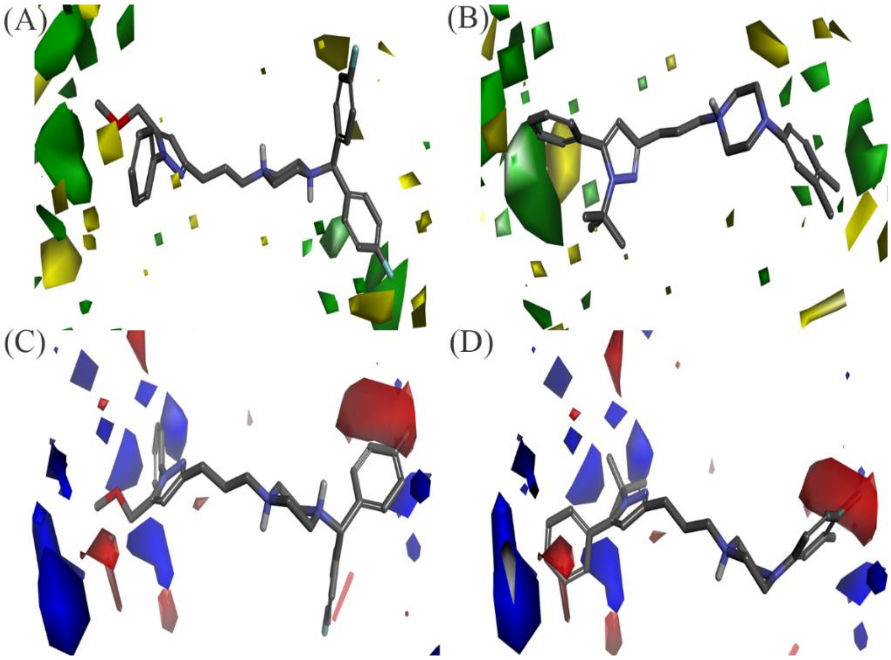

Supplmentary Figure S4. The 3D QSAR model for activity of D4 subtype with compound 103 (A and C), and 5 (B and D). The green contour indicates regions where sterically bulky group increase an inhibitory activity, while the yellow contours indicate regions where sterically bulky groups decrease an inhibitory activity. The blue contour indicates regions where electropositive charged groups increase an inhibitory activity, while the red contour represent where electronegative charged groups improve an inhibitory activity.

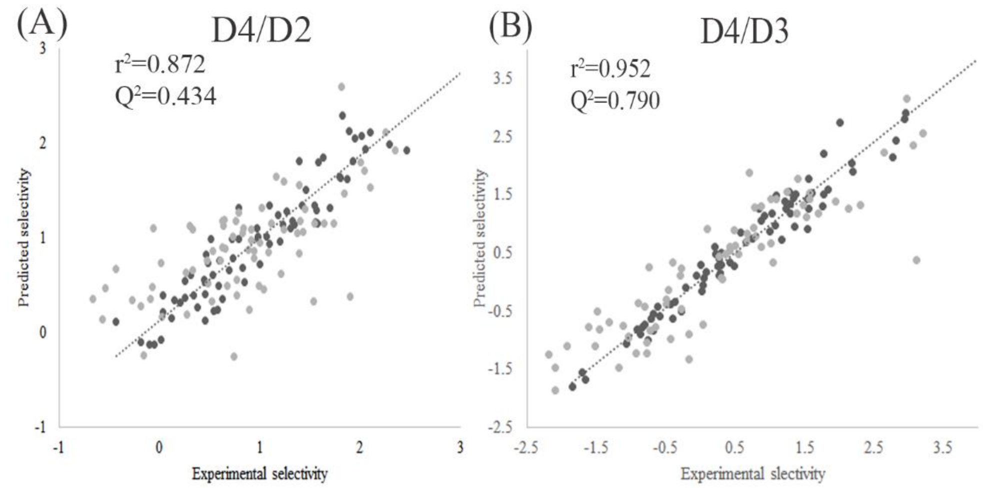

Supplementary Figure S5. Correlation plots between the experimental selectivity and predicted selectivity values of 3D QSAR models for D4/D2 (A) or D4/D3 (B) selectivity. ●: Training set; ●: The test set

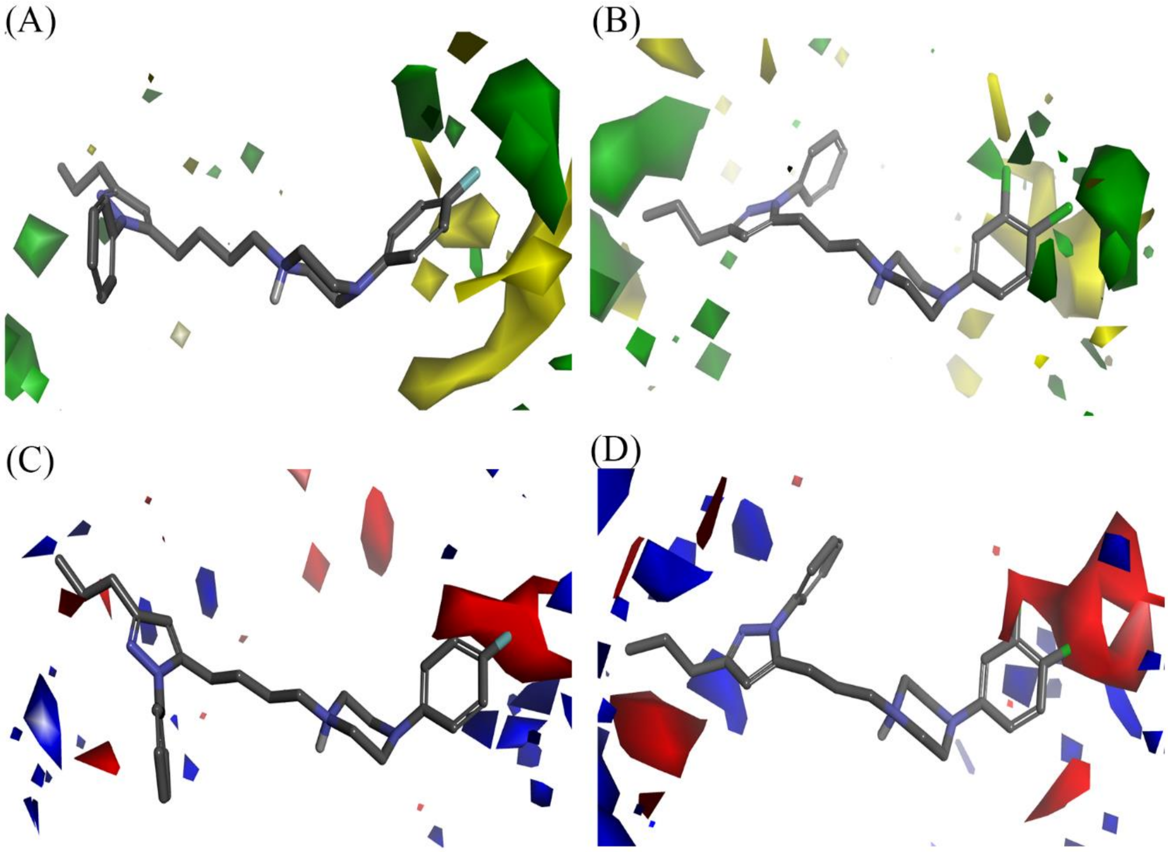

Supplementary Figure S6. The contour maps of 3D QSAR models with D4-selective compounds. The steric and electrostatic contour map are shown together with highly D4-selective compound **46** (A, B) for D4/D2 model and compound **9** (C, D) for D4/D3 models. The green and yellow contours indicate that bulky group is favored and disfavored for D4 selectivity, respectively. The blue contour show areas where electropositive charged groups enhance selectivity for D4 subtype, while red contours represent where electronegative charged groups improve the D4 selectivity.
